# The science of variation in gratitude: demographic variable-based variations in gratitude among Indians

**DOI:** 10.3389/fpsyg.2026.1801344

**Published:** 2026-08-17

**Authors:** Pragya Kasana, Naval Garg, Freda Van Der Walt

**Affiliations:** 1 Delhi Technological University, Delhi, India; 2 Netaji Subhas University of Technology, Delhi, India; 3 Central University of Technology Free State, Bloemfontein, South Africa

**Keywords:** age, demographic analysis, gender, gratitude, *Kṛtagyatā*

## Abstract

Gratitude plays a vital role in human well-being, influencing emotional resilience, social relationships, and overall life satisfaction. Yet its cultural and demographic dimensions remain underexplored. This study assesses gratitude levels using the Hindu Gratitude Scale (HGS) across five dimensions which are parents, God, teachers, fellow human beings, and ecosystem and examines demographic variations in gratitude expression selected through purposive sampling for diverse inclusion of population with a sample size of 2,920. The study investigates how age, gender, education, marital status, occupation, income, and family structure shape gratitude expression in individuals. Findings reveal older adults show higher gratitude than middle-aged individuals. Also, people living in rural area exhibits greater gratitude than urban populations. As per as education qualifications are concerned, gratitude of respondents decrease with their education level. Married individuals show greater gratitude than separated individuals. Occupational and income differences also emerge, with government employees and students expressing more gratitude than private-sector workers and freelancers. The extended and joint family structure showed a higher level of gratitude than the nuclear. The results also found that there is no variation in level of gratitude on the basis of gender. This study explores the level of gratitude in the Indian context, contrasting with Western individualistic perspectives. Insights are useful for further cultural studies, framing inclusive educational policies, and workplace well-being programs. Future research should explore cross-cultural and longitudinal trends in gratitude in terms of diversified population and its evolving nature with each individual.

## Introduction

Gratitude is a multidimensional concept and interpreted differently in various disciplines including psychology, philosophy, religious or spiritual, socio-cultural, anthropological, neuro-scientific and biological. It is regarded not just an emotion, but also as a moral value, a habit, a mindset and a personality trait (Emmons and Shelton, 2002; Wood et al., 2010; Algoe and Stanton, 2012; Garg, 2018). Researchers have also described it as an emotion, a moral virtue, a religious value, an attitude, a personality trait, a habit, a life orientation, and a coping mechanism (Emmons and Shelton, 2002; Wood et al., 2010; Algoe and Stanton, 2012; Garg, 2018). Emmons and McCullough (2003, p. 337) define gratitude as “an emotion, an attitude, a moral virtue, a habit, a personality trait, or a coping response”. As an emotional experience, gratitude arises when one receives something of value from another (Emmons and McCullough, 2003). As a trait, it reflects an individual’s general tendency to recognize and respond with gratitude to the benevolence of others in their positive life events (McCullough et al., 2008, p. 112). When practiced regularly, gratitude becomes a habit, signifying a consistent appreciation for the good aspects of life. As a mood, it is a short-term feeling of thankfulness directed toward a benefactor.

Extensive research has demonstrated the positive associations of gratitude with a range of well-being indicators. Studies have linked gratitude with enhanced subjective well-being (Emmons and Shelton, 2002; Watkins, 2012; Garg et al., 2019), increased life satisfaction (Duckworth et al., 2005; Seligman et al., 2006), and stronger social relationships (Algoe and Stanton, 2012; Algoe et al., 2008). Gratitude is associated with better physical health outcomes (Emmons and McCullough, 2003), improved psychological well-being (Emmons and McCullough, 2003; McCullough et al., 2008; Wood et al., 2010), and meaningful interpersonal relationships (Fredrickson, 2012; Wood et al., 2010). It further contributes to positive psychological, physiological, and social functioning across age groups and settings, including children, adults, and educational environments (Froh et al., 2009; Emmons, 2012; Froh et al., 2011; Layous and Lyubomirsky, 2014). Gratitude is also linked to resilience and optimal functioning (Froh et al., 2011), better emotional regulation (Emmons and McCullough, 2003; Froh et al., 2009), greater happiness and health (Fredrickson, 2012; Fredrickson and Joiner, 2002), effective problem-solving (Fredrickson and Branigan, 2005), reduction in negative emotions (Fredrickson et al., 2000), and enhanced coping with stress (Tugade and Fredrickson, 2004).

However, despite these insights, limited attention has been paid to how gratitude may vary across demographic variables. Even no such very recent studies have done in western also to see demographic variations in level of gratitude. Studying demographic variables in relation to gratitude is essential to understand how different factors like age, gender, income, culture and education shape individuals’ emotional experiences and expressions of thankfulness. The gap is evident in Indian context, where despite the country’s immense sociocultural and religion diversity with different belief system very few empirical works has been conducted to for assessment of level of gratitude on Indian population. These variables offer insight into how cultural, social, and psychological influences that may impact gratitude levels. Exploration of gratitude helps identify specific groups that may benefit from gratitude-based interventions. It also contributes to building more inclusive and context-sensitive models of gratitude, especially in diverse populations. While gender-based differences in gratitude have been widely studied often suggesting that women are more grateful than men (Froh et al., 2014; Kashdan et al., 2009) many aspects are still unfold about the association of gender difference in different country and area. Age has also been explored in relation to gratitude, with some studies suggesting gratitude increases with age due to emotional regulation strategies and prioritization of meaningful experiences (Charles and Carstensen, 2010). However, how gratitude shifts at different life stages still requires deeper investigation. Furthermore, variables such as income level, work experience, and occupational role are very much understudied despite their potential to influence an individual’s dependency, access to support, and perceived self-efficacy all of which affect gratitude (Weiner, 1985). By incorporating these overlooked dimensions, this study adds depth to the psychological understanding of gratitude. Additionally, gratitude in the western models often emphasizes individual emotion and personal well-being, whereas Indian conceptualization is rooted in spiritual duty (*Rṇas*) and collective obligations which view gratitude as an ethical and social responsibility (Garg, 2023). These cultural differences significantly affect how gratitude is experienced and expressed, reinforcing the need to study gratitude through culturally embedded lenses. Western connotation of gratitude is secular; however, the Indian notion of gratitude is ingrained in moral, religious and spiritual philosophies, customs, and practices. Also, gratitude is purposely practiced in daily routine through values system, rituals, festivals, and cultural traditions among Indians. Therefore, it is pertinent to study the variation in level of gratitude across different demographic variables in a non-western setting.

## Literature review

### Gratitude (Kṛitagyatā) from Indian culture perspective

Gratitude, or *Kṛtagyatā* in Sanskrit, is not merely an emotional response in Indian culture but it is a deeply embedded moral and spiritual obligation, reflecting the broader Indian worldview where emotional expressions are intertwined with duties (*dharma*) and sacred commitments (*Rṇas*). In this tradition, gratitude is more than feeling thankful, it is about acting upon one’s indebtedness to others, nature, teachers, and the divine, forming the foundation for social harmony and spiritual progression (Garg, 2023). The philosophical framework of gratitude in Hinduism is primarily structured around the concept of *Pancha Mahāyajñas*, which outlines five sacred debts or duties (*Rṇas*) every individual is born with. These include Pitra Yajña (gratitude toward parents and ancestors), Manuṣya Yajña (duty toward fellow human beings), Deva Yajña (devotion to divine forces), Bhūta Yajña (care for nature and all sentient beings), and Brahma Yajña (reverence for teachers and knowledge systems). These duties, mentioned in Vedas and the Upanishads, lay the moral and spiritual groundwork for gratitude as a way of life. For instance, the oft-quoted Sanskrit verse *“Mātṛdevo bhava, Pitṛdevo bhava”* underscores the sanctity of parents, equating them to deities. Scriptural texts like the Bhagavad Gītā are rich with expressions of gratitude. In Chapter 9, Verse 26 (*patraṁ puṣpaṁ phalaṁ toyaṁ yo me bhaktyā prayacchati.*), the Gītā emphasizes that even the simplest offerings made with devotion are accepted by the divine, illustrating that gratitude lies in the intent rather than the materiality of offerings. Similarly, in Verse 10.41 (*yad yad vibhūtimat sattvaṁ.*), the text teaches that all accomplishments are a reflection of divine grace, urging individuals to remain humble and thankful for every blessing. Gratitude toward teachers (gurus) is considered a sacred duty. The verse *“Guru Brahma, Guru Vishnu, Guru Devo Maheshwara.”* reflects the divine status accorded to educators in Indian thought. This aligns with *Brahma Rṇa*, emphasizing the importance of lifelong learning and acknowledging those who illuminate one’s path to truth. Likewise, reverence for nature is powerfully expressed in Gītā’s Verse 3.12, which states that one who consumes resources without returning them through sacrifice is akin to a thief, reinforcing sustainability and reciprocity with the natural world.

Beyond classical texts, contemporary research has begun to explore gratitude within Indian socio-cultural settings. Examined gratitude’s role in temple attendance, noting that ritualistic offerings often serve as expressions of emotional indebtedness to deities for blessings received. Garg et al. (2021) investigated the influence of Hindu values like *Kṛtagyatā* in organizational behavior, linking it to enhanced workplace cooperation and ethical functioning. Similarly, Garg et al. (2023) validated the Existential Gratitude Scale in the Indian context, confirming its association with spiritual development and subjective well-being.

### Demographic-based variation in gratitude

#### Gender

Research on gender differences in gratitude suggests that men and women may experience and express gratitude differently, although findings are not entirely consistent. Women are generally found to be more emotionally expressive and aware of their feelings, which may contribute to higher gratitude (Simon and Nath, 2004). For instance, Kashdan et al. (2009) reported that women experience and express gratitude more openly, while men may perceive it as uncomfortable or linked to obligation. Similar patterns are observed in studies showing higher gratitude and well-being associations among females (Froh et al., 2009) and higher empathy and gratitude levels among women (Agnieszka et al., 2020). These differences are often explained through gender role socialization, where women are encouraged to be more relational and emotionally expressive, while men are expected to be more restrained (Eagly and Karau, 2002). At the same time, emotional ambivalence may influence how gratitude is expressed (Chen et al., 2012), and cultural context also plays a role, as gender can shape how gratitude relates to broader outcomes across settings (Lasota and Shekhar, 2024). Overall, while women tend to report higher gratitude, these differences are influenced by social roles, emotional processes, and cultural context. Based on above literature, the following hypothesis proposed:

*H1*: Females reports higher level of gratitude than male.

#### Age

Research on age differences in gratitude suggests that gratitude varies across the life span, although findings are not entirely consistent. Empirical studies generally indicate that older individuals tend to report higher levels of gratitude compared to younger and middle-aged groups. For instance, Chopik et al. (2019) found that gratitude was highest among older adults and lowest among younger individuals, while also showing that it’s positive association with well-being remains stable across age groups. Similarly, Allemand and Hill (2016) reported an increase in gratitude from early adulthood to old age. However, some studies highlight inconsistencies, suggesting that age-related differences in gratitude may depend on measurement approaches and sample characteristics (Allemand et al., 2021). Overall, the literature suggests a general tendency for gratitude to increase with age, while also indicating that these patterns are not entirely uniform. On the basis of above literature following will be the hypothesis:

*H2*: Gratitude differs significantly across different age groups.

#### Marital status

There are limited direct studies examining gratitude across marital status as a demographic variable; however, a substantial body of research highlights its importance within marital and romantic relationships. Empirical evidence shows that gratitude strengthens emotional bonds and relationship satisfaction among couples (Gordon et al., 2011; Leong et al., 2019). It also acts as a mechanism through which couples manage stress and enhance relationship quality (Roth et al., 2024), and mediates the link between attachment and relationship satisfaction (Vollmann et al., 2019). At a broader level, recent cross-national evidence also indicates that married individuals tend to report higher levels of gratitude compared to other groups, although these differences may vary across cultural contexts (Okuzono et al., 2025). Overall, while direct comparisons across marital status remain limited, existing findings suggest that relationship context plays an important role in shaping gratitude experiences.

*H3*: Relationship status plays an important role in shaping gratitude experiences, with married individuals reporting higher levels of gratitude than other groups.

#### Level of education

Level of education plays an important role in shaping gratitude both directly and through early socialization process. Empirical evidence suggest that educational exposure, especially in school settings, helps in developing gratitude by promoting prosocial behavior, emotional understanding and better coping skills among students (Bono et al., 2022). Individuals who receive such intervention during childhood tend to develop stronger abilities to handle stress and build positive relationships compared to others. At family level parental education also influence how gratitude and appreciation are nurtured in children. For instance, Chen et al. (2025) found that higher parental education is associated with more effective socialization practices, which in turn support the development of appreciation and gratitude in children. On a broader level, cross-national findings indicate that individuals with higher levels of education tend to report higher gratitude, although these patterns may vary across culture context (Okuzono et al., 2025). Overall, the literature suggests that education, both formal and familial plays a meaningful role in shaping gratitude by influencing learning environments, socialization practices and coping abilities. Based on the above literature the following hypothesis is proposed:

*H4*: Level of education plays a significant role in shaping gratitude, with individuals having higher education reporting higher levels of gratitude.

#### Types of occupation

There are limited direct studies examining how occupation as a demographic variable influences gratitude; however, several studies have explored gratitude within different occupational contexts. Empirical evidence suggests that gratitude plays an important role in employee well-being, job satisfaction, and engagement across professions. For instance, gratitude-based interventions have been shown to improve employee engagement, psychological capital, and overall well-being in organizational settings (Harty et al., 2025). In healthcare contexts, gratitude has been linked with a stronger sense of meaning in life among nurses (Wang et al., 2025) and has also been found to reduce stress and enhance professional satisfaction among clinicians (Caragol et al., 2022). Similarly, among teachers, gratitude is associated with lower burnout and higher work engagement, highlighting its role in managing job demands (Nicuță et al., 2023). Although these studies focus on specific professions rather than occupation as a category, they indicate that work environment, job roles, and occupational stress can influence how gratitude is experienced and expressed. Overall, existing evidence suggests that occupation may play a meaningful role in shaping gratitude, even though direct comparisons across occupational categories remain limited. Based on the above literature following hypothesis is proposed:

*H5*: Occupation plays a significant role in shaping gratitude, with difference in gratitude level across occupational groups.

#### Level of income

There are limited direct studies examining the relationship between income level and gratitude; however, some empirical evidence provides indirect insights. Research suggests that income is associated with emotional experiences, particularly self-regard emotions, which may influence how individuals perceive and express gratitude (Tong et al., 2025). At the same time, studies focusing on low-income populations indicate that gratitude can play a supportive role in maintaining mental health and well-being, especially in challenging economic conditions (Tong et al., 2025). This suggests that individuals with fewer financial resources may rely more on gratitude as a coping mechanism. Additionally, within household contexts, gratitude has been found to vary based on perceived contributions and division of labor, which are often linked to economic roles and financial stability (Daminger et al., 2025). Overall, while direct evidence is limited, existing findings suggest that income level may influence gratitude through differences in life circumstances, emotional experiences, and coping mechanisms. Based on the above literature following hypothesis is proposed:

*H6*: Income level plays a significant role in shaping gratitude, with differences in gratitude levels across income groups.

#### Type of family

There are limited direct studies examining family type as a demographic variable in relation to gratitude; however, existing research highlights the important role of family environment in shaping gratitude. Empirical evidence suggests that a positive family atmosphere significantly influences the development of prosocial behavior, with gratitude acting as a key mediating factor (Li and Li, 2022). Similarly, gratitude has been found to enhance family functioning and overall well-being among parents, indicating its role in strengthening family relationships (Nelson-Coffey and Coffey, 2024). In caregiving and sibling contexts, gratitude is also linked with perceptions of care, contribution, and emotional exchange within the family (Amaro and Miller, 2016). Although these studies do not directly compare different family structures, they suggest that family dynamics, interactions, and levels of connectedness can influence how gratitude is experienced and expressed. Overall, existing evidence indicates that family context plays a meaningful role in shaping gratitude. From the following literature the hypothesis proposed is as follows:

*H7*: Type of family plays a significant role in shaping gratitude, with differences in gratitude levels across different family structures.

#### Type of area

While direct evidence linking type of area (rural vs. urban) with gratitude remains limited, some studies provide indirect insights through related constructs such as materialism and emotional experiences. Research suggests that materialism is often negatively associated with well-being and can influence how individuals experience positive emotions like gratitude (Roberts et al., 2015). In this context, environmental differences become relevant, as exposure to urban settings has been linked with higher materialistic tendencies, whereas more natural or rural environments are associated with lower materialism (Joye et al., 2020). Additionally, studies comparing urban and rural populations have found differences in emotional responses and perceptions, indicating that contextual environments can shape emotional experiences (Zheng et al., 2019). Although these studies do not directly measure gratitude, they suggest that differences in lifestyle, environment, and value orientation across rural and urban areas may influence how gratitude is experienced. Overall, existing evidence indirectly points toward the role of locality in shaping gratitude. According to the above literature following hypothesis is proposed:

*H8*: Type of area (rural vs. urban) plays a significant role in shaping gratitude, with differences in gratitude levels across rural and urban populations.

### Theoretical conceptualization

The present study is grounded in Social Ecological Theory, which explains that human behavior and emotional experiences are shaped by multiple layers of social and environmental contexts (Bronfenbrenner, 1994). In this perspective, emotions such as gratitude are not only individual psychological states but are influenced by interactions within family, workplace, and broader societal environments. Accordingly, demographic variables such as gender and age represent individual-level characteristics, while marital status and family type reflect immediate relational contexts. Similarly, education, occupation, and income capture socio-economic conditions, and locality (rural–urban) represents the broader environmental setting. Together, these variables provide a comprehensive framework to understand how gratitude is shaped across different layers of social life. While other theories such as the Broaden-and-Build Theory (Fredrickson, 2012) focus on the outcomes of positive emotions and the Find-Remind-and-Bind Theory (Algoe, 2012) emphasizes interpersonal bonding through gratitude, they do not adequately explain how diverse social and demographic conditions influence the experience of gratitude. Therefore, Social Ecological Theory is considered more appropriate for the present study, as it offers a holistic and integrated framework to examine how gratitude varies across interconnected personal, relational, and environmental contexts.

## Research methodology

### Sampling and data collection

The study employed a purposive sampling technique to obtain a heterogeneous sample comprising respondents from diverse demographic backgrounds. A total of 3,130 respondents residing in the Delhi-National Capital Region (NCR), India, participated in the survey. Delhi-NCR was deliberately selected as the study site due to its high demographic diversity and cosmopolitan character. As one of India’s largest metropolitan regions, Delhi attracts substantial internal migration from across different states, resulting in a population that varies widely in terms of age, income, education, language, religion, and cultural backgrounds. According to the Census of India (2011) and subsequent population estimates, Delhi hosts a population exceeding 16 million, with a significant proportion comprising migrants, thereby making it a microcosm of India’s socio-cultural diversity. This diversity makes Delhi-NCR an appropriate and strategically relevant setting for examining demographic variations in gratitude. Furthermore, the region is supported by extensive urban infrastructure that facilitates access to a wide cross-section of society. Notably, the Delhi Metro, one of the largest urban transit systems globally, records average daily passenger journeys exceeding 5 million, reflecting its role as a major mobility network connecting individuals from varied socioeconomic and occupational groups (Delhi Metro Rail Corporation, 2024). This makes metro stations particularly suitable locations for accessing a heterogeneous population. Accordingly, data were collected during August–September 2024 by approaching respondents at multiple metro stations across and individuals were approached at different time slots to ensure diversity in responses, and participation was entirely voluntary. The inclusion criteria required participants to be aged 18 years or above and currently residing in the Delhi-NCR region. Exclusion criteria included incomplete responses, questionnaires with excessive missing values, and entries exhibiting inconsistent or patterned responses (e.g., straight-lining). In addition, participants were recruited through an online seminar on gratitude to further enhance sample reach. To ensure data quality and minimize duplication, responses were screened for identical patterns and unusually short completion times, and online submissions were restricted to one response per device/IP where feasible. After removing incomplete and inconsistent responses, a final sample of 2,920 valid cases was retained for analysis. The adequacy of the sample size is supported by Cochran’s (1977) recommendation of a minimum of 384 respondents for large heterogeneous populations at a 95% confidence level.

### Measures

Gratitude was measured using Hindu Gratitude Scale (HGS) developed and validated by Garg (2023). It comprised 15 items rated on a five-point rating scale ranging from 1 (strongly disagree) to 5 (strongly agree). The scale is based on Indian *Rṇas* conceptualization of gratitude. *Rṇas* are sacred obligations that every individual is expected to fulfill during their lifetime. The scale captures five dimensions of gratitude, i.e., appreciation for fellow human beings; appreciation for family, ancestors, and cultural values; appreciation for God; appreciation for knowledge, skills and talents; and appreciation for the ecosystem. Although the scale is named as the ‘*Hindu Gratitude Scale*,’ its focus on universal values such as respect for teachers, appreciation of nature and acknowledgement of social bonds make it broadly applicable to people of all faiths and religions. The Scale ensures that the measurement of gratitude is not limited to a single religious perspective but is inclusive of the shared ideological heritage found across Indian cultures. The five dimensions of the scale are inherently secular as they are not confined to any single religious narrative and instead capture a holistic perspective of gratitude. In India, gratitude is deeply embedded in cultural practices and moral values across various communities, irrespective of religious affiliations. Therefore, the scale’s multidimensional approach aligns with the Indian worldview which sees gratitude not merely as a religious sentiment but as an ethical and social obligation. This universal applicability in perspective makes it a suitable and effective tool for evaluating gratitude across diverse samples within the Indian demographic landscape. A few sample items are “I feel grateful to my parents for this beautiful life,” “A lot of people have contributed positively in my life,” and “I am thankful to all the teachers and mentors who have imparted knowledge and skills to me.”

### Data analysis

The reliability and convergent validity of the scale was assessed using Cronbach’s alpha, composite reliability, and Average Variance Explained (AVE) estimates. The discriminant validity was evaluated with the help of the Fornell and Larcker (1981) testing method. To ensure the independence of five dimensions of gratitude, the Variance Inflation Factor (VIF) values were examined to check for multicollinearity. Factor loadings represent the extent to which each item correlates with its respective construct, ensuring the relevance and representativeness of the items. This statistical analysis is performed using IBM SPSS Statistics (Version 25). The factorial validity of the scale was verified with the help of the confirmatory factor analysis (CFA) administered in the AMOS 16.0. In addition, t-test and ANOVA (Analysis of Variance) were administered to examine the demographics-based variations across five dimensions of gratitude. We also used polynomial regression to analyze the variation in gratitude across different age groups. Specifically, we utilized a cubic polynomial model to capture the non-linear relationship between gratitude and age. This approach allows for a more nuanced understanding of how gratitude changes with age of the respondents. The cubic regression model was selected based on preliminary analyses that indicated a non-linear trend in the data. The coefficients obtained from the regression provide insights into how changes in age are associated with variations in gratitude levels.

## Results

### Demographics, descriptive statistics, reliability, validity and multicollinearity

Table 1 presents the demographic profile of the respondents across key variables. In addition to categorical classification, age was also examined as a continuous variable to provide a more comprehensive description of the sample. The mean age of the respondents was 35.9 years (SD = 16.4), with an age range of 18 to 75 years. Table 2 reported the descriptive statistics, reliability, and validity estimates of five dimensions of gratitude: appreciation for parents, God, fellow human beings, teachers, and nature. Factor loadings for most items are above 0.7, indicating strong correlations with their respective constructs, with a few exceptions that are still within acceptable ranges. The Cronbach’s Alpha and Composite Reliability values are above the threshold range of 0.70, which concluded reliability of the scale. The Average Variance Extracted (AVE) estimates were above 05.0, which confirmed scales’ convergent validity. Table 3 represents a correlation matrix, wherein diagonal elements of the matrix represent the square root of AVE while off-diagonal values indicate inter-construct correlations. All diagonal values exceeded their respective inter-construct correlations confirming discriminant validity of the scale according to the criterion. The Variance Inflation Factor (VIF) values are below the threshold of 5, which indicates no significant multicollinearity issues among the five dimensions of gratitude. Table 2 also reported acceptable factor loading of all items of the scale. Also, the five-factored model also showed good model with acceptable fit indices as of *χ*^2^/df = 2.950, Comparative Fit Index (CFI) = 0.96, the Non-Normed Fit Index (NNFI) = 0.96, the Standardized Root Mean Square Residual (SRMR) = 0.07, and the Root Mean Square Error of Approximation (RMSEA) = 0.047. These results reconfirmed the reliability, factorial, convergent, and discriminate validity of the scale.

**Table 1:** Sample description.

Variable	Category	Frequency	Percentage
Gender	Male	1,435	49.14
	Female	1,485	50.86
Marital Status	Single	1,530	52.40
	Married	1,305	44.69
	Separated or divorced	85	2.91
Education Level	High school	665	22.77
	Graduate	705	24.14
	Post graduate	1,015	34.76
	Doctorate	535	18.32
Occupational Status	Student	885	30.31
	Government job	645	22.09
	Private job	645	22.09
	Business	190	6.51
	Freelancer	555	19.01
Income Level	No income	1,155	39.55
	Rs. 1–Rs. 5,00,000	690	23.63
	Rs. 5,00,001–Rs10,00,000	640	21.92
	Rs. 10,00,001–Rs. 15,00,000	200	6.85
	Above Rs. 15,00,000	235	8.05
Family type	Nuclear family	1,000	34.25
	Joint family	1,320	45.21
	Extended joint family	600	20.55
Locality	Urban	2,510	85.96
	Rural	410	14.04
Age	18–29	1,565	53.60
	30–39	430	14.73
	40–49	305	10.45
	50–59	180	6.16
	60–69	295	10.10
	70 and above	145	4.97

Source: Primary data.

**Table 2:** Descriptive statistics, reliability, and validity estimates.

Dimensions	Items	Loading	Mean	S. D.	CA	CR	AVE	VIF
Appreciation for parents	P1	0.821	4.27	0.68	0.72	0.78	0.55	1.52
	P2	0.805						
	P3	0.679						
Appreciation for god	G1	0.720	4.32	0.70	0.80	0.82	0.60	1.91
	G2	0.809						
	G3	0.799						
Appreciation for fellow human being	FH1	0.718	4.03	0.78	0.79	0.77	0.53	1.63
	FH2	0.818						
	FH3	0.646						
Appreciation for teachers	T1	0.798	4.18	0.69	0.90	0.87	0.68	1.66
	T2	0.841						
	T3	0.839						
Appreciation for ecosystem	N1	0.814	3.97	0.76	0.86	0.86	0.67	1.52
	N2	0.825						
	N3	0.816						

Source: Primary data.

**Table 3:** Correlation matrix and discriminant validity analysis.

Dimensions	1	2	3	4	5
Appreciation for parents	1 **(0.735)**				
Appreciation for god	0.544*	1 **(0.733)**			
Appreciation for fellow human beings	0.432*	0.489*	1 **(0.745)**		
Appreciation for teacher	0.401*	0.482*	0.542*	1 **(0.825)**	
Appreciation for ecosystem	0.309*	0.527*	0.409*	0.464*	1**(0.733)**

Source: Primary data, ** Sig. at 0.05, Square roots of AVE are mentioned diagonally.

### Age-based variation in gratitude

**Figure 1:**
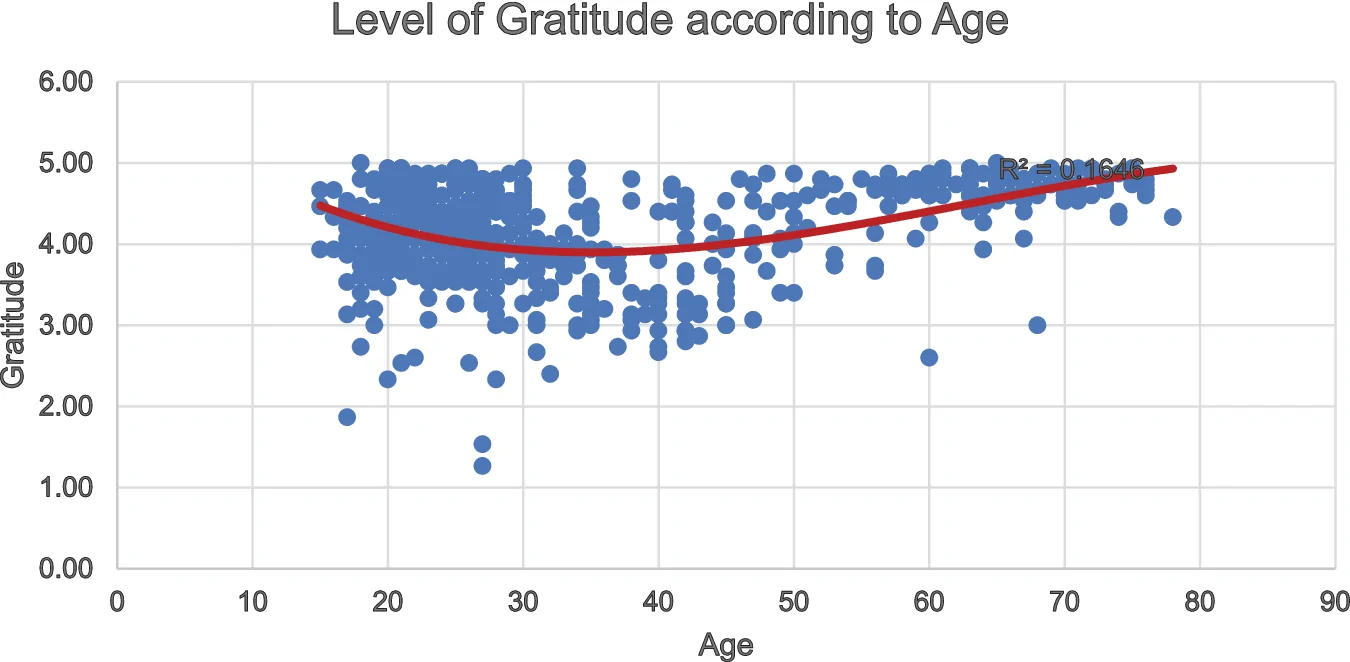
Age-based variations in gratitude. Source: Primary data.

Table 4 showed age-based significant differences in all dimensions of gratitude. Individuals in the youngest age group (18–29) and older age groups (60 and above) reported higher levels of gratitude; however, people in the middle age brackets (30–49) showed comparatively lower levels of gratitude. The Bonferroni correction *Post Hoc* analysis revealed significant differences in gratitude levels across the age’s group. Acknowledging significant age-based variations across all five dimensions of gratitude, we used polynomial regression analysis with a cubic degree to examine the non-linear relationship between age and gratitude (refer to Figure 1). The resulting regression equation is as follows:


$$\text{Gratitude}=0.0000\times {\mathrm{Age}}^{3}+0.0026\times {\mathrm{Age}}^{2}–0.1328\times \mathrm{Age}+5.9234$$

**Table 4:** Age-based variations in gratitude.

Factors	Age	Mean	*f*-value	Effect size	Sig
Appreciation for parents	18–29	4.1	5.32	η^2^*p* = 0.02	0.002*
	30–39	3.8			
	40–49	3.6			
	50–59	3.9			
	60–69	4.2			
	70 and above	4.4			
Appreciation for god	18–29	4.0	4.76	η^2^*p* = 0.02	0.00*
	30–39	3.7			
	40–49	3.5			
	50–59	3.8			
	60–69	4.3			
	70 and above	4.5			
Appreciation for fellow human being	18–29	4.0	6.45	η^2^*p* = 0.03	0.01*
	30–39	3.6			
	40–49	3.4			
	50–59	3.7			
	60–69	4.1			
	70 and above	4.3			
Appreciation for teacher	18–29	3.9	4.12	η^2^*p* = 0.02	0.00*
	30–39	3.5			
	40–49	3.3			
	50–59	3.7			
	60–69	4.0			
	70 and above	4.2			
Appreciation for ecosystem	18–29	4.2	5.98	η^2^*p* = 0.02	0.01*
	30–39	3.7			
	40–49	3.5			
	50–59	3.8			
	60–69	4.2			
	70 and above	4.4			
Overall gratitude	18–29	4.1	6.87	η^2^*p* = 0.03	0.01*
	30–39	3.7			
	40–49	3.5			
	50–59	3.8			
	60–69	4.2			
	70 and above	4.4			

Source: Primary data, * Sig at 0.05.

The coefficients provide valuable insights into how gratitude changes across different age groups. The negative coefficient of the linear term (−0.1328) suggests that gratitude decreases with increasing age during early and middle life stages. However, the positive coefficient of the quadratic term (0.0026) indicates a curvilinear effect where gratitude begins to recover and increase after a certain age. The cubic equation and graph show a U-shaped trend which demonstrates that in an early stage of life, gratitude is high but as the age increases the gratitude starts declining but after certain age gratitude gradually starts increasing. The analysis reveals a significant and interpretable relationship between age and gratitude. The R-squared value of 0.1645 suggests that the model explains approximately 16.45% of the variance in gratitude levels. In essence, the results suggest that gratitude tends to decrease in middle age but begins to increase again as individuals grow older, reflecting a dynamic interplay between life experiences, maturity, and emotional perspective over the lifespan. This highlights the importance of considering age-specific interventions to foster gratitude in different stages of life.

Table 5 reported the results of t-test administered to examine represents gender-based variations in gratitude. The result showed no statistically significant variations among male and female respondents across all five dimensions of gratitude. It also revealed insignificant gender-based variations in overall gratitude (mean_male_ = 4.09, mean_female_ = 4.25, *p* = 0.331). Table 6 highlighted results of ANOVA administered to explore marital status-based variations in gratitude. The result indicated no statistically differences in appreciation toward parents and God among single, married, and separated respondents. However, significant marital status-based variations were reported in appreciation for fellow human beings, teachers, ecosystem, and overall gratitude with married people being most grateful followed by single and separated individuals. The Bonferroni correction *Post Hoc* analysis revealed significant differences in gratitude levels across three marital status groups.

**Table 5:** Gender-based variations in gratitude.

Factors	Gender	Mean	*t*-value	Effect size	Sig.
Appreciation for parents	Male	4.25	1.772	d = 0.08	0.184
	Female	4.30			
Appreciation for god	Male	4.26	0.946	d = 0.05	0.331
	Female	4.37			
Appreciation for fellow human beings	Male	4.01	0.172	d = 0.01	0.678
	Female	4.04			
Appreciation for teacher	Male	4.17	0.785	d = 0.04	0.376
	Female	4.19			
Appreciation for ecosystem	Male	3.95	0.104	d = 0.01	0.747
	Female	3.98			
Overall gratitude	Male	4.09	0.947	d = 0.07	0.331
	Female	4.25			

Source: Primary data.

**Table 6:** Martial-status-based variations in gratitude.

Factors	Marital status	Mean	*f*-value	Size effect	Sig
Appreciation for parents	Single	4.27	1.261	η^2^*p* = 0.01	0.096
	Married	4.29			
	Separated	3.12			
Appreciation for god	Single	4.24	1.238	η^2^*p* = 0.01	0.114
	Married	4.42			
	Separated	3.06			
Appreciation for fellow human being	Single	4.03	3.217	η^2^*p* = 0.02	0.000*
	Married	4.04			
	Separated	3.76			
Appreciation for teacher	Single	4.23	2.872	η^2^*p* = 0.02	0.000*
	Married	4.13			
	Separated	4.10			
Appreciation for ecosystem	Single	3.03	6.824	η^2^*p* = 0.03	0.000*
	Married	3.91			
	Separated	3.88			
Overall gratitude	Single	4.15	4.321	η^2^*p* = 0.02	0.034*
	Married	4.19			
	Separated	3.80			

Source: Primary data, * Sig at 0.05.

Table 7 showed statistically significant differences in all five dimensions and overall gratitude across respondents with different educational credentials. Interestingly, individuals with high school education reported a high level of gratitude followed by graduates, post graduates and doctorates. It suggested that gratitude levels tend to decrease with higher education levels, potentially due to heightened career pressures and competitive environments associated with higher education. The *post hoc* analysis using the Bonferroni correction confirmed the significant variations across four educational groups. Table 8 suggested significant variations across all five dimensions of gratitude based on respondents’ occupation. Individuals in government jobs experienced the highest level of gratitude followed by students, private job employees, business owners and freelancers in all the dimensions. It suggests that safety an individual gets from a government job enhances the feeling of gratitude. The post hoc analysis using Bonferroni correction signifies the same result and showed lowest level of gratitude among freelancers as compared to others.

**Table 7:** Education level-based variations in gratitude.

Factors	Education	Mean	*f*-value	Size effect	Sig
Appreciation for parents	High school	4.56	3.427	η^2^*p* = 0.02	0.001*
	Graduate	4.18			
	Post Graduate	3.97			
	Doctorate	3.45			
Appreciation for god	High school	4.68	4.871	η^2^*p* = 0.02	0.000*
	Graduate	4.53			
	Post Graduate	4.35			
	Doctorate	4.19			
Appreciation for fellow human being	High school	4.57	8.310	η^2^*p* = 0.03	0.000*
	Graduate	4.45			
	Post graduate	3.88			
	Doctorate	3.66			
Appreciation for teacher	High school	4.56	5.123	η^2^*p* = 0.02	0.000*
	Graduate	4.17			
	Post graduate	3.76			
	Doctorate	3.04			
Appreciation for ecosystem	High school	4.50	8.765	η^2^*p* = 0.03	0.000*
	Graduate	3.96			
	Post graduate	3.90			
	Doctorate	3.62			
Overall gratitude	High school	4.54	1.501	η^2^*p* = 0.01	0.001*
	Graduate	4.25			
	Post graduate	3.35			
	Doctorate	3.60			

Source: Primary data, *Sig at 0.05.

**Table 8:** Occupation-based variations in gratitude.

Factors	Occupation	Mean	f-value	Size effect	Sig
Appreciation for parents	Student	4.27	3.163	η^2^*p* = 0.02	0.008*
	Government job	4.44			
	Private job	4.23			
	Business	4.11			
	Freelancer	3.96			
Appreciation for god	Student	4.25	5.763	η^2^*p* = 0.03	0.000*
	Government job	4.46			
	Private job	4.34			
	Business	3.96			
	Freelancer	3.95			
Appreciation for fellow human being	Student	4.10	9.246	η^2^*p* = 0.03	0.000*
	Government job	4.32			
	Private job	3.85			
	Business	3.49			
	Freelancer	3.72			
Appreciation for teacher	Student	4.33	5.607	η^2^*p* = 0.03	0.000*
	Government job	4.30			
	Private job	4.03			
	Business	3.78			
	Freelancer	3.76			
Appreciation for ecosystem	Student	4.03	7.187	η^2^*p* = 0.03	0.000*
	Government job	4.19			
	Private job	3.66			
	Business	3.61			
	Freelancer	3.67			
Overall gratitude	Student	4.20	2.830	η^2^*p* = 0.02	0.016*
	Government job	4.11			
	Private job	4.24			
	Business	3.98			
	Freelancer	3.90			

Source: Primary data, *Sig at 0.05.

Table 9 indicated significant variations in gratitude level across all dimensions based on income levels. The analysis revealed that gratitude was highest among individuals with no income declining progressively with higher income brackets, reaching the lowest levels for individuals earning above Rs. 15,00,000 earners. The post hoc analysis using Bonferroni correction also revealed that lower-income groups (particularly those with no income) express significantly higher gratitude across most dimensions compared to higher-income groups. Table 10 revealed that the type of family structure significantly influences all dimensions of gratitude. Individuals from extended joint families consistently reported the highest levels of gratitude, followed by those in joint families, while nuclear family members showed the lowest levels. The results of the Bonferroni *Post Hoc* analysis further confirmed significant differences between nuclear, joint, and extended joint families, supporting the impact of family structure on gratitude expression. Table 11 showed that in terms of area showed a notable difference in the level of gratitude toward all the dimensions and rural respondents showed higher levels of gratitude toward all the dimensions. The overall gratitude also describes the same pattern which reveals rural individuals are more grateful than urban ones (mean_rural_ = 4.189, mean_urban_ = 4.11, *p* = 0.004).

**Table 9:** Income-based variations in gratitude.

Factors	Income level	Mean	f-value	Size effect	Sig
Appreciation for parents	No income	4.58	2.381	η^2^*p* = 0.02	0.002*
	Rs. 1–Rs. 5,00,000	4.39			
	Rs. 5,00,001–Rs. 10,00,000	4.22			
	Rs. 10,00,001–Rs. 15,00,000	4.11			
	Above Rs. 15,00,000	4.01			
Appreciation for god	No income	4.52	3.905	η^2^*p* = 0.02	0.004*
	Rs. 1–Rs. 5,00,000	4.48			
	Rs. 5,00,001–Rs. 10,00,000	4.30			
	Rs. 10,00,001–Rs. 15,00,000	4.11			
	Above Rs. 15,00,000	4.09			
Appreciation for fellow human being	No income	4.29	8.159	η^2^*p* = 0.03	0.000*
	Rs. 1–Rs. 5,00,000	4.11			
	Rs. 5,00,001–Rs. 10,00,000	3.82			
	Rs. 10,00,001–Rs. 15,00,000	3.84			
	Above Rs. 15,00,000	3.71			
Appreciation for teacher	No income	4.30	5.704	η^2^*p* = 0.03	0.000*
	Rs. 1–Rs. 5,00,000	4.26			
	Rs. 5,00,001–Rs. 10,00,000	4.12			
	Rs. 10,00,001–Rs. 15,00,000	3.85			
	Above Rs. 15,00,000	3.79			
Appreciation for ecosystem	No income	4.17	12.383	η^2^*p* = 0.04	0.000*
	Rs. 1–Rs. 5,00,000	4.03			
	Rs. 5,00,001–Rs. 10,00,000	4.17			
	Rs. 10,00,001–Rs. 15,00,000	3.55			
	Above Rs. 15,00,000	3.44			
Overall gratitude	No income	4.38	7.689	η^2^*p* = 0.03	0.001*
	Rs. 1–Rs. 5,00,000	4.22			
	Rs. 5,00,001–Rs. 10,00,000	4.16			
	Rs. 10,00,001–Rs. 15,00,000	4.09			
	Above Rs. 15,00,000	4.01			

Source: Primary data, *Sig at 0.05.

**Table 10:** Family type-based variations in gratitude.

Factors	Type of family	Mean	f-value	Size effect	Sig
Appreciation for parents	Nuclear family	3.80	1.261	η^2^*p* = 0.01	0.001*
	Joint family	4.22			
	Extended joint family	4.40			
Appreciation for god	Nuclear family	3.67	1.238	η^2^*p* = 0.01	0.000*
	Joint family	4.00			
	Extended joint family	4.30			
Appreciation for Fellow Human being	Nuclear family	3.50	3.217	η^2^*p* = 0.02	0.000*
	Joint family	4.10			
	Extended joint family	4.30			
Appreciation for teacher	Nuclear family	3.40	2.872	η^2^*p* = 0.02	0.000*
	Joint family	3.90			
	Extended joint family	4.20			
Appreciation for ecosystem	Nuclear family	3.70	6.824	η^2^*p* = 0.03	0.000*
	Joint family	4.10			
	Extended joint family	4.44			
Overall gratitude	Nuclear family	3.60	4.321	η^2^*p* = 0.02	0.002*
	Joint family	4.10			
	Extended joint family	4.30			

Source: Primary data, *Sig at 0.05.

**Table 11:** Area-based variations in gratitude.

Factors	Locality	Mean	*f*-value	Size effect	Sig
Appreciation for parents	Urban	4.02	0.611	d = 0.35	0.043*
	Rural	4.58			
Appreciation for god	Urban	4.12	0.004	d = 0.40	0.009*
	Rural	4.67			
Appreciation for fellow human being	Urban	3.78	0.672	d = 0.45	0.004*
	Rural	4.52			
Appreciation for teacher	Urban	3.65	1.410	d = 0.42	0.023*
	Rural	4.36			
Appreciation for ecosystem	Urban	3.94	0.015	d = 0.28	0.009*
	Rural	4.12			
Overall gratitude	Rural	4.18	0.292	d = 0.10	0.004*
	Urban	4.11			

Source: Primary data, *Sig at 0.05.

## Discussion

This study aimed to examine how demographic variables are associated with variations in gratitude within the Indian context. Grounded in Social Ecological Theory (Bronfenbrenner, 1994), the findings are interpreted by considering how different layers of individual, relational, and environmental contexts shape emotional experiences such as gratitude. Overall, the results suggest that gratitude is not uniform but varies across demographic conditions, reflecting the influence of interconnected social environments. The analysis uncovered several meaningful patterns across factors such as gender, age, marital status, education, occupation, income, work experience, and locality.

With respect to gender, the findings indicate no significant difference in gratitude levels between males and females. This result aligns with the Gender Similarity Hypothesis proposed by Hyde (2014), explains that male and females are more similar than different across most psychological traits including gratitude. In Indian context, this result may reflect the relatively uniform transmission of value-based orientations within Indian families, where both males and females are socialized into shared moral frameworks emphasizing respect, duty, and acknowledgment of others. The concept of *dharma* is not gender-exclusive but is embedded in role-based expectations across individuals (Paranjpe, 2013), which may contribute to similar expressions of gratitude.

Secondly, the age-based analysis revealed an inverted U-shaped pattern, where gratitude is relatively higher among younger and older individuals and lower during middle age. This variation may reflect differences in life roles and responsibilities, as midlife is often associated with increased career and family pressures that may limit opportunities for emotional reflection. In the Indian philosophical context, life is understood through stages associated with different duties and orientations (Mathew, 2025). The *Brahmacharya* stage (youth) emphasizes learning and dependence on parents and teachers, which may foster higher gratitude. In contrast, the *Grihastha* stage (middle age) involves greater responsibility and self-reliance, where expressions of gratitude may become less salient. In later stages such as *Vanaprastha* and *Sannyasa*, individuals tend to shift toward reflection, detachment, and valuing relationships and life experiences more deeply, which may again enhance gratitude. While this framework provides a meaningful cultural lens, these interpretations remain indicative, as the underlying processes were not directly measured.

Thirdly the findings based on marital status reveals that separated or divorced individuals report lower levels of gratitude compared to single and married individuals except in gratitude toward parents and God. The reason behind this is the emotional and physical toll of separation as studies show that divorced individuals often experience higher stress, loneliness and poorer mental health (Sbarra and Whisman, 2022). Financial and social instability post separation can also contribute to emotional strain (Paul et al., 2020) and personality shifts like reduced agreeableness and emotional stability may further impact gratitude level (Spikic et al., 2021). Since gratitude is an emotional response, it can be weakened when individuals are facing personal hardships or negative emotions. In the Indian context, these effects may be even more prominent due to the deeply rooted cultural view of marriage as a sacred and lifelong union, not merely a legal contract.

The result of variation in gratitude on the basis of education level showed gratitude level of an individual consistently decrease as education level increase especially for individuals holding advanced degrees (Post graduates and Doctorates) compared to those with lower education level (High school and graduate). High qualification and learning lead to increase in more analytical and critical thinking of an individual which leads to less relying on emotional reasoning. Studies suggest that higher education is associated with the development of traits such as self-efficacy, autonomy, and achievement orientation (Puerta Gómez et al., 2024; Thørrisen and Sadeghi, 2025), which may shift individuals toward internal attributions of success rather than external acknowledgment, thereby influencing expressions of gratitude. At the same time, early educational experiences play an important role in fostering gratitude, as school-based interventions have been shown to enhance emotional understanding, prosocial behavior, and coping abilities (Bono et al., 2022). Additionally, parental education influences how gratitude and appreciation are socialized during childhood, with higher parental education linked to more structured and effective socialization practices (Chen et al., 2025). These findings suggest that while education contributes to the development of gratitude in early stages, higher levels of education may also encourage independence and self-reliance, which could influence how gratitude is experienced and expressed.

The findings indicate that students and government employees reported relatively higher levels of gratitude compared to individuals in private, freelance, and business roles. This pattern may be understood in terms of differences in work context, stability, and daily experiences. Empirical evidence shows that gratitude is positively associated with employee well-being, engagement, and psychological resources in structured work environments (Harty et al., 2025). Similarly, in professions such as healthcare and teaching, gratitude has been linked with a stronger sense of meaning, reduced stress, and better coping with job demands (Wang et al., 2025; Caragol et al., 2022; Nicuță et al., 2023). In this context, government employment, which is often characterized by relatively higher job security and stability, may provide a consistent and supportive environment that facilitates positive emotional experiences such as gratitude. In contrast, individuals in freelance and business roles may face greater uncertainty, fluctuating income, and higher performance pressures, which may influence how gratitude is experienced. Students, being more dependent on external support systems such as family and educational institutions, may also have more opportunities to experience and express gratitude.

Individuals with moderate incomes show more gratitude than respondents with more income. This may be because individuals with moderate and lower-income levels many times depend on their parents and external factors as compared to the higher income level person because they are more independent and think that whatever they achieve in their life with their own efforts and hard work. In a study of Stellar et al. (2018) it was revealed that individuals with limited financial resources tend to exhibit greater compassion to those with higher financial resources. Similarly, another study of Paul et al. (2020) revealed that people with fewer financial resources engage in higher levels of prosocial behavior, such as helping others and donating to charity than their wealthier counterparts. This aligns with findings from Puente-Díaz and Meixueiro (2016), which showed that higher income levels are often associated with lower expressions of gratitude. Their study highlights that gratitude is inversely related to financial independence and self-reliance, with individuals in moderate income brackets showing higher levels of gratitude and altruism than wealthier individuals.

The analysis based on family structure shows that individuals from extended joint families report the highest levels of gratitude across all dimensions. This may be due to the collectivist nature of Indian culture, where extended families live together, share responsibilities, and follow moral and spiritual teachings that promote thankfulness (Banerjee, 2024; Shin et al., 2020). On the other hand, people from nuclear families showed the lowest gratitude levels, possibly because of a more individualistic lifestyle and fewer opportunities for shared emotional experiences. In smaller family units, values like mutual respect and appreciation may not be reinforced as regularly. Those living in joint families also demonstrated relatively high levels of gratitude. This is likely because joint families still promote togetherness, shared duties, and intergenerational bonding, which help develop a sense of gratitude from daily interactions and support.

When it comes to locality and area of an individual the findings reveal that rural area individuals are more thankful in all the dimensions as compared to the urban area individuals. In rural India, the upbringing and value system are significantly different from urban contexts. People in villages often grow up deeply connected to their roots, shaped by a collectivist culture that prioritizes community over individuality. Life in these areas is guided not just by social norms but by a sense of duty toward family, society, and even the environment. The moral framework is strongly influenced by the concept of *dharma* (righteous duty), which encourages respect for elders, communal harmony, and gratitude for natural resources. They have lower exposure to materialism and individualistic thinking. Dixit and Sinha (2024), in their cross-cultural study, found that such collectivist orientations in Indian culture significantly enhance expressions of gratitude. This embedded system of shared responsibility and interdependence contributes to a stronger sense of appreciation for every small movement in daily life.

## Future implications

### Theoretical implications

The present study is grounded in Social Ecological Theory (Bronfenbrenner, 1994), which posits that individual experiences and behaviors are shaped by multiple, interconnected layers of social and environmental contexts. By examining gratitude across demographic variables such as gender, age, marital status, education, occupation, income, family structure, and locality, the study extends this theoretical perspective by demonstrating how gratitude varies across different ecological layers. The findings suggest that gratitude is not solely an individual emotional disposition but is associated with broader relational and contextual conditions, thereby reinforcing the applicability of Social Ecological Theory in understanding emotional constructs such as gratitude. Furthermore, this study contributes to theory development by providing empirical evidence from a non-Western context, highlighting the importance of incorporating cultural and contextual variability within ecological frameworks. The observed patterns indicate that gratitude may operate differently across social environments, offering a basis for future research to examine the mechanisms through which ecological factors influence emotional experiences. In addition, the use of the Hindu Gratitude Scale (HGS) strengthens the theoretical contribution by capturing gratitude as a culturally embedded construct rather than a purely universal emotion. This supports the need for context-sensitive measurement approaches and provides a foundation for future research to further explore culturally grounded conceptualizations of gratitude within ecological frameworks.

### Practical implications

This study offers valuable practical implications for policy design and interventions across various sectors. It provides insights into employee management by revealing no significant gender differences in gratitude levels, suggesting that organizations can implement uniform policies for male and female employees, fostering inclusivity and fairness. Additionally, the findings highlight lower gratitude levels among divorced individuals, possibly due to emotional resilience challenges and societal judgments. This insight allows companies and social organizations to develop support systems, counseling, and community-building initiatives tailored to this demographic. The Google hiring process not only prioritizes technical skills but also cultural fit, often referred to as “Googleyness.” This includes attributes like problem-solving ability, adaptability, and a collaborative mindset which help Google to reduce employee turnover and no problem of quite quitting. The initiative by Salesforce to established Employee Resource Groups (ERGs) to support employees from various demographic backgrounds, including those based on gender, race, sexual orientation provides community, mentorship, and advocacy, helping to ensure that policies and workplace practices are responsive to the unique needs of different employee groups. The study also emphasizes the importance of gratitude in educational settings. It shows that individuals pursuing advanced degrees have lower levels of gratitude, indicating a need for more mental health awareness programs and courses apart from studies to enhance student well-being, foster positive peer relationships, and improve academic performance. In the workplace, understanding occupational differences in gratitude can inform employee engagement programs and mindfulness practices, promote a positive organizational culture and increasing job satisfaction. Furthermore, the study suggests targeted interventions for groups with lower gratitude levels, such as middle-aged individuals and those in private or freelance occupations, to enhance emotional resilience and overall well-being. Policymakers and social organizations can leverage these findings to design community programs that promote gratitude as a cultural and spiritual value, thereby enhancing social harmony and communal well-being. These implications offer a comprehensive approach to integrating gratitude into social policies, educational frameworks, and organizational practices, contributing to a more positive and inclusive society.

### Limitations, future directions and conclusion

Despite addressing a significant gap in this research by assessing the level of gratitude in Indian context, the present study carries its own limitations. Although India provides a rich cultural and spiritual backdrop for studying gratitude, the absence of religious affiliation as a demographic variable restricts deeper interpretation and future studies should incorporate religious identity to better understand how gratitude varies across spiritual traditions such as Hinduism, Islam, Christianity, Jainism, Buddhism and others. Moreover, this study can be done on more sample size and in different areas of the country as the size of the sample is disproportion and can induce sampling bias. The study employed a cross-sectional design which captures a gratitude in a single point of time but many studies suggest gratitude evolves with life stages, personal growth and experience so future research would benefit from longitudinal designs to explore the stability and change. Cross-cultural studies cam also done by attaching different gratitude scale for a robust result in diverse population. Overall, this study provides valuable insights into the patterns of gratitude with Indian context highlighting important demographic study. These findings not only deepen our understanding of gratitude but also offer practical guidance for fostering well-being and inclusivity across diverse sectors of society.

## Data Availability

The raw data supporting the conclusions of this article will be made available by the authors, without undue reservation.
